# The Impact of Different Dietary Ratios of Soluble Carbohydrate-to-Neutral Detergent Fiber on Rumen Barrier Function and Inflammation in Dumont Lambs

**DOI:** 10.3390/ani14111666

**Published:** 2024-06-02

**Authors:** Shufang Li, Tian Ma, Yawen An, Yu Zhang, Xiaodong Yang, Aiwu Gao, Hairong Wang

**Affiliations:** 1 Animal Nutrition and Feed Science, Inner Mongolia Agricultural University, Hohhot 010018, China; lishufang0325@163.com (S.L.);; 2 Food Science, Inner Mongolia Agricultural University, Hohhot 010018, China

**Keywords:** soluble carbohydrate-to-neutral detergent-fiber ratio, rumen epithelium, tight junctions, inflammation, NF-κB /MAPK, Dumont lambs

## Abstract

**Simple Summary:**

The rumen is a digestive organ in ruminants that is affected by soluble carbohydrate (SCHO) levels in the diet. This study confirmed that as the proportion of SCHO to NDF in the diet increased, the rumen pH decreased, the LPS concentration increased, and the papilla height and area increased. When the ratio of SCHO to NDF was 2.0, the tight junctions were disrupted, inflammatory cytokines levels were increased, and NF-κB/MAPK was activated.

**Abstract:**

Appropriate soluble carbohydrate (SCHO)-to-NDF ratios in the diet are essential for rumen health. The effects of different SCHO-to-NDF ratios (1.0, 1.5, and 2.0) on rumen barrier function and inflammation in Dumont lambs (n = 18, 6 replicates per treatment) was investigated. The SCHO:NDF ratio was altered by replacing the forage (*Leynus chinensis*) with corn grain. With an increase in the proportion of SCHO, the final body weight (FBW), average daily gain (ADG), soluble carbohydrate intake (SCHOI), and LPS level increased; and the neutral detergent fiber intake (NDFI), ruminal papillae height, papillae area, and pH decreased (*p* < 0.05, *p*_lin_ < 0.05). The medium CHO:NDF group had increased claudin-1 mRNA (*p* < 0.05, *p*_lin_ = 0.005, *p*_quad_ = 0.003) and protein (*p* < 0.05, *p*_quad_ < 0.001) levels; the high CHO:NDF group had increased occludin mRNA and protein (*p* < 0.05, *p*_lin_ = 0.001) levels. The level of the anti-inflammatory cytokine IL-10 was significantly greater in the medium CHO:NDF group than in the high CHO:NDF group (*p* < 0.05, *p*_quad_ < 0.001). With an increase in the ratio of SCHO, the mRNA level and concentration of the proinflammatory cytokines IL-1β, IL-6, and TNF-α linearly increased (*p* < 0.05, *p*_lin_ < 0.05), and those in the high CHO:NDF group were significantly greater than those in the low CHO:NDF group. The levels of phosphorylated p65 (*p*_lin_ = 0.003), IκB-α (*p*_lin_ < 0.001), and JNK (*p*_lin_ = 0.001) increased linearly, and those in the high CHO:NDF group were significantly greater than those in the other two groups (*p* < 0.05). Therefore, when the SCHO-to-NDF ratio was increased to 1.5, the rumen epithelium was not affected, but when the ratio was increased to 2.0, NF-κB and MAPK were activated in the rumen epithelium, leading to impaired barrier function and inflammation. The suitable NFC:NDF ratio for the short-term fattening of Dumont lambs was found to be 1.50.

## 1. Introduction

Maximizing animal production efficiency to achieve the greatest economic benefits while ensuring animal health and the quality and safety of livestock products is a key objective in livestock production. Roughage is a source of NDF, which stimulates rumination and gastrointestinal peristalsis [1]. Cereal grains, as the main source of soluble carbohydrates, are used as primary ingredients in concentrates with the aim of increasing energy levels and digestibility in the growing–finishing diets of ruminants [2]. The rumen is the main site of nutrient digestion and metabolism in ruminants, and it is affected by the ratio of concentrate to roughage in the diet. The rumen buffering capacity and volatile fatty acid (VFA) absorption in ruminants are correlated with the carbohydrate fermentation rate, with the pH in the rumen remaining within the normal range of 6.5–7.5 [3]. When ruminants are fed a large amount of concentrate, rumen peristalsis slows, and the rumen epithelium shows cuticle shedding and vacuolization [4], which affects the absorption of VFAs, resulting in elevated levels of VFAs and lactic acid and a decrease in pH [5,6]; these changes can induce subacute gastric acidosis (SARA) in severe cases [7]. Moreover, a diet high in soluble carbohydrate leads to rumen microbiota disorders, increased levels of bacterial endotoxins and biogenic amines, and impaired tight junctions between rumen epithelial cells, which affects rumen epithelial barrier function [8,9,10]. In addition, when ruminants are fed a high-concentrate diet, LPS produced in the rumen can be transferred into blood or gastrointestinal epithelial cells through the paracellular pathway and transcellular transport and is recognized by epithelial cells or immune cells [11], which activate NF-κB and MAPK via the LPS/TLR4 pathway, leading to rumen or systemic inflammation [12,13].

The Inner Mongolia region is the largest pasture area in China, and herders usually graze their sheep with a small amount of supplemental energy and protein feed, such as corn and soybean meal; this diet pattern is low-energy and low-nitrogen and cannot satisfy the growth demand of hybrid meat sheep. However, in the mainstream commercial goat market, many grain starch-based total mixed rations (TMRs) and pellet feeds are added to the diet to improve productivity, which increases the incidence of nutritional metabolic diseases and can damage the digestive tract of animals [14]. Therefore, special attention should be given to rumen health when shifting from a low-concentrate diet to a high-concentrate diet. Thus, in the present study, Dumont lambs were used to study the effects of low-, medium-, and high-SCHO:NDF-ratio diets on the morphology of the rumen mucosa epithelium tissue, rumen barrier function, and inflammation in lambs. Taken together, these findings reveal the mechanism by which diet structure influences the health of the rumen of lambs and provide a theoretical basis for the healthy fattening of lambs.

## 2. Materials and Methods

### 2.1. Animal Feeding and Experimental Design

Eighteen healthy male Dumont lambs (25.73 ± 2.17 kg BW, 3 mo. age) were used to evaluate the effect of treatment on growth performance, rumen pH, rumen lipopolysaccharide (LPS) content, papillae morphology, and inflammatory modulators. Lambs were individually housed and fed diets with different SCHO:NDF ratios of 1.0 (L group), 1.5 (M group), or 2.0 (H group). The SCHO:NDF ratio was altered by replacing the forage (*Leynus chinensis*) with corn grain (Table 1). According to the NY/T 816-2004 China Mut-ton Sheep feeding standard, the experiment lasted 75 days, with a 15-day adaptation period and a 60-day formal period. These lambs were randomly assigned to a metabolic cage (1.5 × 1 × 1 m) in a sheep house and fed twice a day at 08:00 and 18:00, with free access to feed and water.

### 2.2. Growth Performance and Feed Intake

The crude protein, calcium, phosphorus, ADF, and NDF levels were determined according to the feed analysis and feed detection method described by Zhang [15]; the SCHO level was determined according to the method described by Huang [16]. Each lamb was weighed on an empty stomach on day 1 and day 60 of the official period of the trial, and daily feed intake was recorded. The ADG, AGFI, SCHOI, NDFI, and F/G were subsequently calculated.

### 2.3. Sample Collection

At days 50, 51, and 52, rumen fluid was sampled 0 h, 2 h, 4 h, 6 h, 8 h, and 10 h after feeding through a rubber stomach tube with a vacuum pump and filtered through four-layer gauze immediately after pH was measured with a pH meter (OHAUS Corporation, Parsippany, NJ, USA). The rumen was washed with PBS after the animals had been sacrificed once the experiment ended. Approximately 4 cm^2^ of rumen ventral sac tissue was collected and fixed using a 4% paraformaldehyde solution for histological analysis. Five grams of rumen epithelial tissue was also collected and stored at −80 °C.

### 2.4. Determination of LPS Levels in the Rumen Epithelium

The rumen epithelium was centrifuged at 3000× *g* for 20 min, and the supernatant was carefully collected. The LPS concentration was determined using an ELISA kit according to the manufacturer’s instructions (Wuhan Genemy Biotechnology Co., Ltd., Wuhan, China).

### 2.5. Histological Analysis

Hematoxylin–eosin-stained sections were prepared according to the methods described by Dai et al. [17] and Gui [18], with some modifications. The 4% paraformaldehyde-fixed rumen epithelium was paraffin-embedded after gradual dehydration with different concentrations of ethanol, and 6 μm sections were cut via a paraffin microtome (Leica RM2235; Leica Microsystems, Wetzlar, Germany), spread on slides, and stained with hematoxylin–eosin after hydration. After the sections were sealed with neutral gum, they were observed under an orthostatic microscope (Nikon ECLIPSE Ni; Nikon Corporation, Tokyo, Japan), and images were captured and analyzed using image-acquisition software (Image View 4.1; Century Kexin Scientific Instrument Co., Ltd., Beijing, China). Five fields of view were selected for each section, and the three longest papillae under the field of view were utilized to determine the height, width, and area of the rumen papillae.

### 2.6. RNA Analysis

Total RNA was extracted from rumen tissues using TRIzol reagent (Takara Corporation, Otsu, Japan). After approximately 0.1 g of rumen epithelium was removed and ground in liquid nitrogen, chloroform fractionation, isopropanol precipitation, and ethanol washing were performed to obtain pure total RNA. The concentration and purity of total RNA were assessed using an Implen P330 NanoPhotometer (Implen, Germany) to ensure that the 260/280 ratio in all the samples was between 1.8 and 2.0, after which, the RNA was transcribed to cDNA. The sequences of primers used are provided in Supplementary Table S1. PCR amplification was performed using a Light Cycler 480 real-time PCR system (Roche, Switzerland) under the following conditions: pre-denaturation at 95 °C for 30 s; 40 cycles of 95 °C for 5 s and 60 °C for 20 s; melting at 95 °C for 5 s, 60 °C for 60 s, and 95 °C for continuous acquisition; and cooling at 50 °C for 30 s. The relative mRNA expression of the target genes was calculated by the 2^−ΔΔCt^ method with β-actin as an internal reference.

### 2.7. Cytokine Content Analysis

A total of 0.1 g of rumen epithelium was homogenized using a hand-held glass homogenizer, and 0.9 mL of PBS was added. The supernatant was collected carefully after centrifugation at 3000× *g* for 20 min. The concentrations of inflammatory cytokines (IL-1β, IL-6, TNF-α, IL-10, and IFN-γ) were determined using ELISA reagent according to the manufacturer’s instructions (Wuhan Genemy Biotechnology Co., Ltd., Wuhan, China).

### 2.8. Western Blot Analysis

Approximately 0.1 g of rumen epithelium was added to 1 mL of Western protein isolation buffer (Beyotime, Shanghai, China) and fully homogenized using a hand-held glass homogenizer. Protein samples were adjusted to a concentration of 50 μg/20 μL using a BCA protein concentration kit (Beyotime). The amount of protein that was loaded in each gel well was 40 μg, and the proteins were transferred to a polyvinylidene difluoride (PVDF) membrane (Pall Corporation, Washington, NY, USA) after SDS–PAGE by a TE22 Mighty Small Transfer (Hoefer Inc., Holliston, MA, USA). Subsequently, the PVDF membrane was blocked in a non-protein-blocking solution (Shanghai Sangon Biotech Co. Ltd., Shanghai, China) for 1 h at room temperature, the primary antibody was applied, and the membrane was incubated at 4 °C overnight. The PVDF membrane was washed three times with 1× Tris-buffered saline containing Tween 20 (TBST) for 5 min each time. Then, the sections were incubated with the fluorescent secondary antibody for 1 h at room temperature in the dark and washed with TBST three times for 10 min each. Information on the antibody types and dilution ratios is listed in Supplementary Table S2. The signals were scanned on an Odyssey DLx imaging system (LI-COR Inc., Lincoln, NE, USA), and the imaging results were analyzed using Image Studio Version 5.2 software (LI-COR Inc., USA).

### 2.9. Statistical Analysis

The data were analyzed using one-way ANOVA in SPSS 23 (IBM Corp., New York, NY, USA), followed by Duncan’s post hoc test and linear and quadratic trend fitting. The results are presented as the mean ± SEM, with significance levels set at *p* < 0.05.

## 3. Results

### 3.1. Growth Performance and Feed Intake

As shown in Table 2, the medium CHO:NDF group had an increased AGFI (*p* < 0.05, *p*_quad_ = 0.002). Polynomial contrast analysis revealed that with an increase in the NFC:NDF ratio in the diet, the FBW (*p* < 0.05, *p*_lin_ < 0.010, *p*_quad_ = 0.018), ADG (*p* < 0.05, *p*_lin_ < 0.050, *p*_quad_ = 0.012), and SCHOI (*p* < 0.05, *p*_lin_ = 0.001, *p*_quad_ = 0.002) showed linear and quadratic increases, with the medium and high CHO:NDF groups showing greater increases than the low CHO:NDF group (*p* < 0.05); the NDFI (*p* < 0.05, *p*_lin_ < 0.001, *p*_quad_ = 0.006) showed linear and quadratic decreases, with that of the high CHO:NDF group being lower than that of the low and medium CHO:NDF groups (*p* < 0.05).

### 3.2. Changes in pH and LPS Concentration in the Rumen

**Figure 1:**
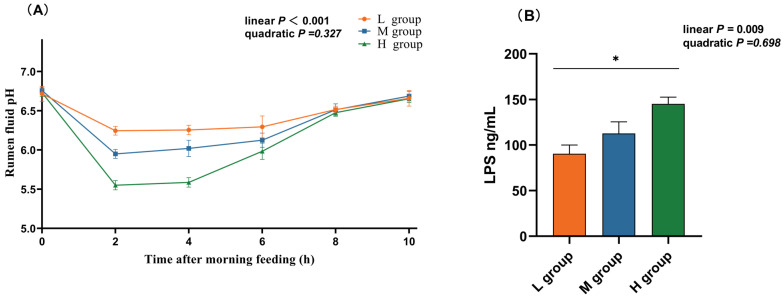
Effect of different soluble carbohydrate-to-neutral detergent fiber ratios in diets on pH (**A**) of rumen fluid and LPS (**B**) concentration of rumen epithelium. Data were expressed as mean ± SEM. * *p* < 0.05 mean significant differences. L group = SCHO-to-NDF ratio was 1.0; M group = SCHO-to-NDF ratio was 1.5; H group = SCHO-to-NDF ratio was 2.0.

As the proportion of SCHO to NDF in the diet increased, the ruminal pH linearly decreased (*p*_lin_ < 0.05), and in the high-SCHO:NDF group, the rumen fluid pH significantly decreased compared to that in both the low- and medium-SCHO:NDF groups 2 h and 4 h after morning feeding (*p* < 0.05, Figure 1A). The high-SCHO:NDF group also had significantly greater LPS concentrations than the low-SCHO:NDF group did, and the LPS levels increased linearly as the SCHO:NDF ratio increased (*p*_lin_ < 0.05, Figure 1A).

### 3.3. Morphological Analysis of the Rumen Epithelium

**Figure 2:**
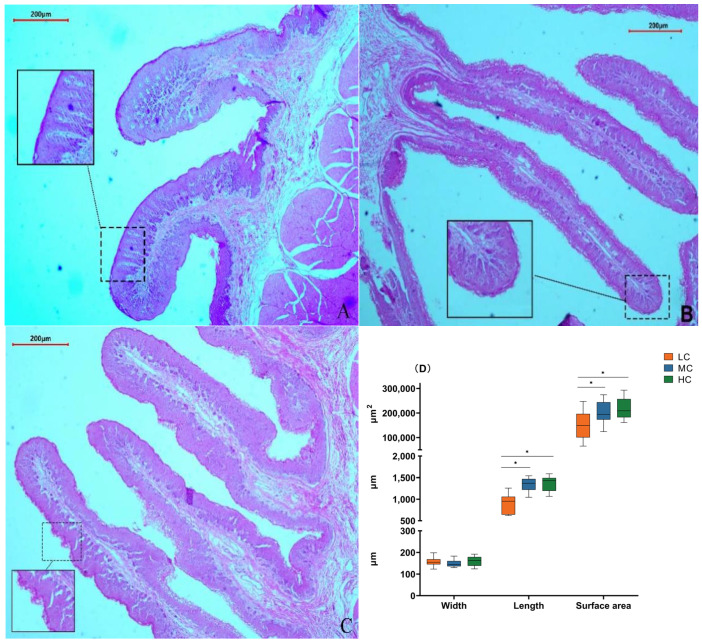
Effect of different soluble carbohydrate-to-neutral detergent fiber ratios in diets on rumen epithelial morphology of lambs. Error lines represent maximum and minimum values. (**A**) Morphology of rumen papillae in the L group; (**B**) morphology of rumen papillae in the M group; (**C**) morphology of rumen papillae in the H group. (**D**) Morphological statistics of rumen papillae. * *p* < 0.05 mean significant difference. L group = SCHO-to-NDF ratio was 1.0; M group = SCHO-to-NDF ratio was 1.5; H group = SCHO-to-NDF ratio was 2.0.

We found that in the low-SCHO:NDF group, the surface of the rumen papillae was smooth without noticeable shedding (Figure 2A). The thickness of the rumen papilla cuticle slightly increased in the medium-SCHO:NDF group (Figure 2B). However, the rumen epithelial cuticle thickness increased in the high-SCHO:NDF group, and rumen papillae were notably enlarged and irregular in shape (Figure 2C). Furthermore, polynomial analyses revealed that papillae height followed a linear (*p*_lin_ < 0.001) and quadratic (*p*_quad_ < 0.001) pattern of increase with increasing levels of dietary SCHO:NDF, and papillae area followed a linear (*p*_lin_ < 0.001) pattern of increase, with the high-SCHO:NDF group having significantly greater papillae height and area than the low- and medium-SCHO:NDF groups (*p* < 0.05). The papillae width did not significantly differ among the three groups (*p >* 0.05) (Figure 2D, Table S3).

### 3.4. Rumen Epithelium Tight Junction mRNA and Protein Expression

**Figure 3:**
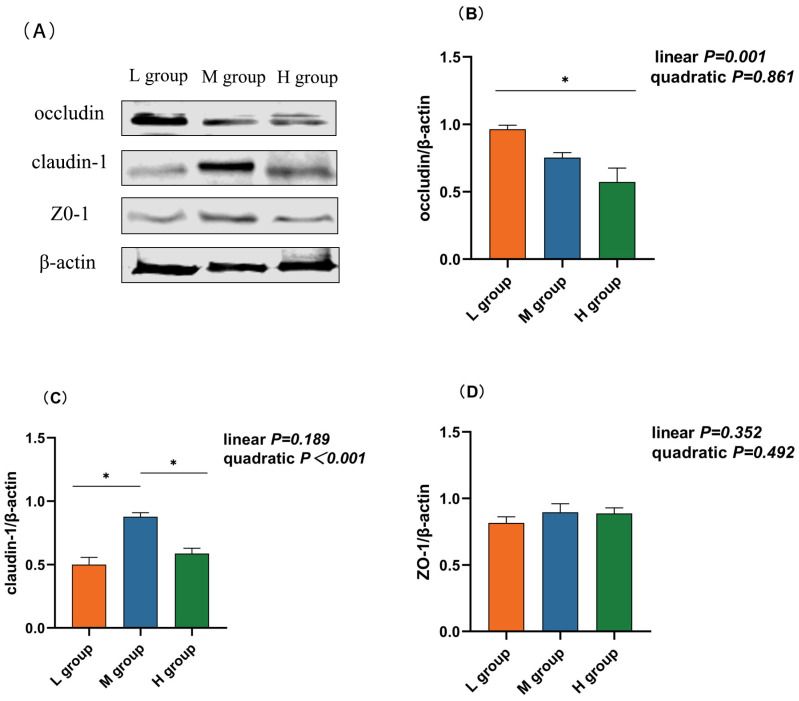
Effect different soluble carbohydrate-to-neutral detergent fiber ratios in diets on the protein abundance of rumen epithelium (**B**) occludin, (**C**) cloudin-1, and (**D**) ZO-1. (**A**) Representative bands from Western blot analysis. Data were expressed as mean ± SEM. In the same row, values with different letter superscripts mean significant difference (*p* < 0.05). * *p* < 0.05 mean significant differences. L group = SCHO-to-NDF ratio was 1.0; M group = SCHO-to-NDF ratio was 1.5; H group = SCHO-to-NDF ratio was 2.0.

As the proportion of SCHO to NDF in the diet increased, the occludin mRNA and protein expression levels linearly decreased (*p*_lin_ = 0.001) and were significantly lower in the high-SCHO:NDF group than in the low-SCHO:NDF group (*p* < 0.05, Table 3 and Figure 3C). The claudin-1 mRNA level increased and then decreased in a linear (*p*_lin_ = 0.021) and quadratic (*p*_quad_ = 0.003) manner, and the claudin-1 mRNA expression in the medium-SCHO:NDF group was significantly greater than that in the low-SCHO:NDF group (*p* < 0.05). The protein abundance was also significantly greater in the medium group than that in the low- and high-SCHO:NDF groups (*p* < 0.05) (Figure 3, Table 3).

### 3.5. Rumen Epithelium Inflammatory Factor mRNA and Protein Expression

Fluorescence quantitative PCR was used to detect changes in the mRNA expression of inflammatory factors in the rumens of lambs fed diets supplemented with different soluble carbohydrate-to-neutral detergent fiber ratios. As presented in Table 4, polynomial analyses revealed that the IL-10 mRNA level increased and then decreased quadratically (*p*_quad_ < 0.001) with increasing SCHO:NDF ratios in the diet, and the IL-10 level in the medium-SCHO:NDF group was significantly greater than that in the low- and high-SCHO:NDF groups (*p* < 0.05). The mRNA expression of IL-1β (*p*_lin_ = 0.008), IL-6 (*p*_lin_ = 0.011), and TNF-α (*p*_lin_ = 0.003) increased linearly, while the expression of IL-1β and TNF-α in the high-SCHO:NDF group was significantly greater than that in the low-SCHO:NDF group (*p* < 0.05); the expression of IL-6 in the medium- and high-SCHO:NDF groups was significantly greater than that in the low-SCHO:NDF group (*p* < 0.05).

### 3.6. Inflammatory Factor Concentrations in the Rumen Epithelium

**Figure 4:**
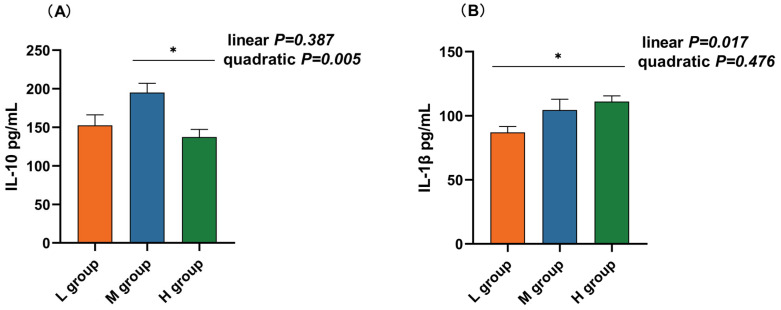

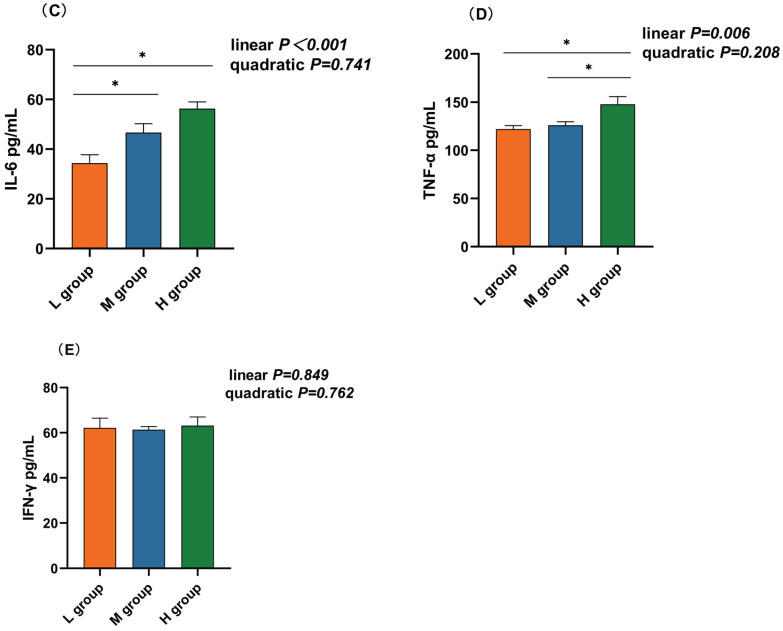
Effect of different soluble carbohydrate-to-neutral detergent fiber ratios in diets on rumen epithelium inflammatory factors: (**A**) IL-10, (**B**) IL-1β, (**C**) IL-6, (**D**) TNF-α, and (**E**) IFN-γ concentrations. Data were expressed as mean ± SEM. * *p* < 0.05 mean significant differences. L group = SCHO-to-NDF ratio was 1.0; M group = SCHO-to-NDF ratio was 1.5; H group = SCHO-to-NDF ratio was 2.0.

The rumen IL-10 concentration in the medium-SCHO:NDF (195.00 pg/mL) group was significantly greater than that in the high-SCHO:NDF (137.41 pg/mL) group (*p* < 0.05, Figure 4A). Polynomial analyses revealed that the level of IL-10 increased and then decreased quadratically (*p*_quad_ = 0.005) and that the levels of IL-1β (*p*_lin_ = 0.017), IL-6 (*p*_lin_ < 0.01) and TNF-α (*p*_lin_ = 0.006) increased linearly with increasing SCHO:NDF levels in the diet. The IL-1β concentration was significantly greater in the high-SCHO:NDF (111.01 pg/mL) group than in the low-SCHO:NDF (86.98 pg/mL) group (*p* < 0.05, Figure 3B). The IL-6 concentration in the high-SCHO:NDF (56.30 pg/mL) group was significantly greater than that in the low-SCHO:NDF (34.41 pg/mL) group, and that in the medium-SCHO:NDF (46.69 pg/mL) group was also significantly lower than that in the low-SCHO:NDF (34.41 pg/mL) group (*p* < 0.05, Figure 4C). Furthermore, the concentration of TNF-α was significantly greater in the high-SCHO:NDF (147.76 pg/mL) group than in the low- and medium-SCHO:NDF (122.00 and 125.96 pg/mL) groups (*p* < 0.05, Figure 4D).

### 3.7. Abundance of Proteins in the NF-κB/MAPK Pathway

**Figure 5:**
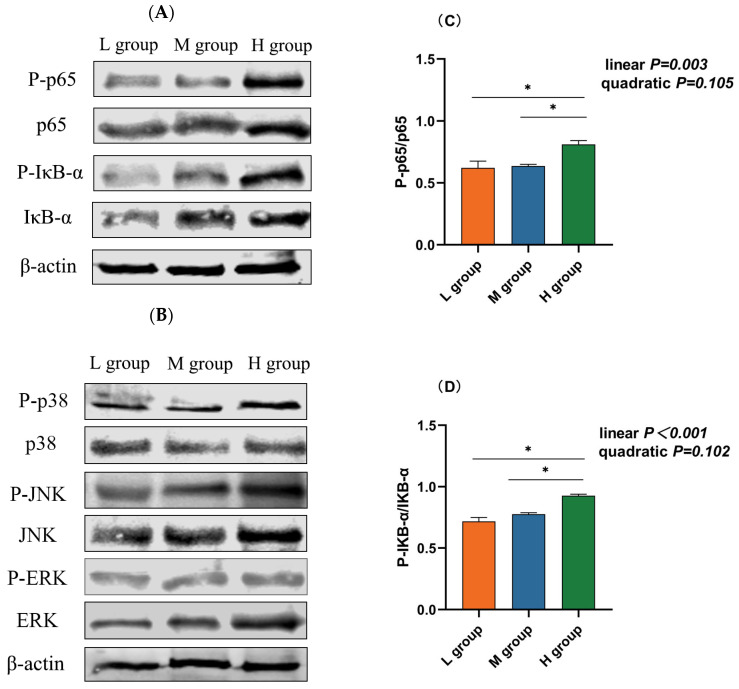

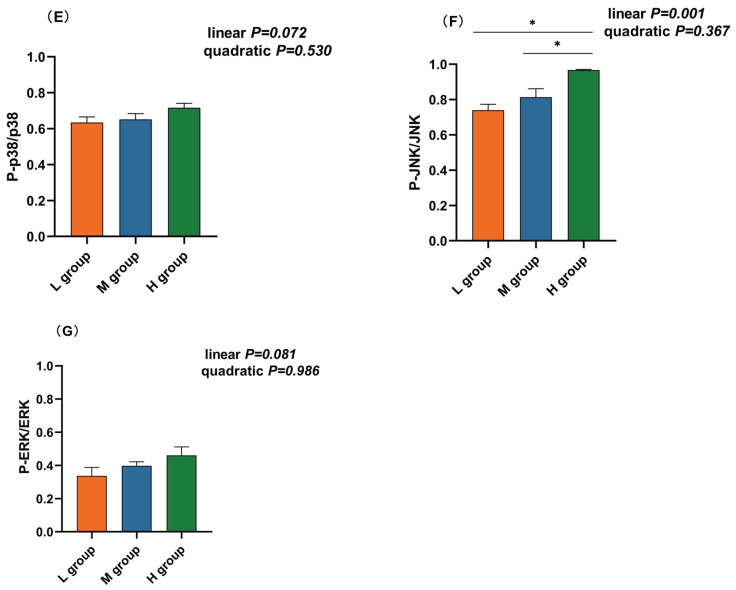
Effect of different soluble carbohydrate-to-neutral detergent fiber ratios in diets on phosphorylation levels of (**C**) p65 and (**D**) IκB-α, (**E**) p38, (**F**) JNK, and (**G**) ERK in the rumen epithelium. (**A**,**B**) Representative bands from Western blot analysis. Data were expressed as mean ± SEM. * *p* < 0.05 mean significant differences. L group = SCHO-to-NDF ratio was 1.0; M group = SCHO-to-NDF ratio was 1.5; H group = SCHO-to-NDF ratio was 2.0.

The NF-κB and MAPK pathways are vital inflammatory pathways. Western blotting revealed that the abundance of phosphorylated p65 (*p*_lin_ = 0.003), IκB-α (*p*_lin_ < 0.001), and JNK (*p*_lin_ = 0.001) increased linearly with an increase in the ratio of SCHO to NDF in the diets, and the levels of phosphorylated p65, IκB-α, and JNK in the group with a high SCHO:NDF ratio were significantly greater than those in the low- and medium-SCHO:NDF groups (*p* < 0.05, Figure 5C,D,F). There was no significant difference in the phosphorylation levels of proteins between the low- and medium-SCHO:NDF groups (*p* > 0.05, Figure 5E,G).

## 4. Discussion

Growth performance is an important quantitative parameter that is used to measure animal development and nutrition. In the present study, we showed that the growth performance of lambs increased significantly with an increase in the dietary SCHO:NDF ratio, but a high SCHO:NDF ratio did not result in greater weight gain, which may have been a result of reduced feed intake in the high-SCHO:NDF group compared to the medium-SCHO:NDF group. Rumen pH plays a crucial role in maintaining homeostasis of the internal environment [3], and stability is closely related to the nutritional level of feed. When the fiber level in the ration is too low or the starch content is too high, the rumen rapidly ferments to produce a large amount of VFA and lactic acid, and the pH decreases [5,19,20]. We found that after morning feeding, the rumen pH tended to decrease and then increase, and the rate of decrease in the rumen pH increased with an increasing SCHO:NDF ratio. This result was consistent with the findings of Kennelly et al. [21] and Han et al. [22]. The rumen pH of lambs in the high-SCHO:NDF group was considerably lower than that in the low-SCHO:NDF group at 2 and 4 h after morning feeding. However, this value did not reach the standard for SARA, which is usually defined as a rumen pH < 5.6 lasting at least 3 h [23,24,25]. This result could have been due to the oral sampling method, which caused saliva to mix into the rumen fluid and increased the pH.

A normal rumen morphology is a fundamental prerequisite for ensuring the proper digestion and absorption of nutrients. The composition of diets could influence both the structural and functional aspects of the rumen epithelium [4,26]. The rational composition of diets, along with high-quality roughage, could mitigate symptoms such as the aggregation and keratinization of rumen papillae and reduce the incidence of ruminal digestive disorders attributed to feed [27]. In the present study, we revealed that the height and area of the rumen papillae increased linearly with increasing SCHO:NDF ratios. In addition, irregularities and shedding of rumen papillae were observed in the high-SCHO:NDF group. This study is consistent with previous research conducted using Hu sheep [28], goats [29], and calves [30] fed diets with different SCHO-to-NDF ratios. This consistency suggested that a high SCHO-to-NDF ratio in the diet affects rumen morphology and disrupts barrier function.

Tight junctions (TJs) are intercellular connections that are located in the apical part of the epithelium, and they are vital in the formation of the mucosal barrier [31,32]. Occludin and claudins are two essential transmembrane proteins that make up TJs, together with the ZO protein family, and form scaffolds [32]. The loss or enrichment of tight junction proteins is closely associated with various pathological conditions [33,34,35]. Claudin-1 regulates paracellular permeability, and its overexpression improves barrier function [36]. Occludin, which was identified as the first transmembrane protein of TJs, plays a pivotal role in maintaining the integrity of TJ barriers. Our findings indicated that the medium-SCHO:NDF group exhibited significantly elevated claudin-1 levels in the rumen epithelium. Conversely, occludin expression was significantly lower in the high-SCHO:NDF group than in the low-SCHO:NDF group, consistent with the results of previous studies of Zhang et al. [37], Ma et al. [29], and Liu et al. [38]. In addition, previous studies have shown that a high SCHO-to-NDF ratio in the diet reduces occludin expression in the jejunum [39] and colon [40].

Previous studies have shown that a high SCHO-to-NDF ratio in the diet influences tight junctions in several ways. On the one hand, a high SCHO-to-NDF ratio in the diet decreases the rumen pH, thereby increasing the LPS concentration [17]. LPS induces excessive NO production and modifies TJ protein expression and subcellular localization [41]. On the other hand, a high SCHO-to-NDF ratio in the diet can reduce the expression of rumen epithelial tight junction proteins through the MAPK pathway [37]. Furthermore, previous studies have confirmed that inflammatory cytokines regulate tight junction protein expression and distribution [42]. TNF-α increases the paracellular permeability of the intestinal epithelium, reduces epithelium transepithelial resistance, and disrupts tight junction distribution by interfering with assembly and disassembly mechanisms. This experiment illustrated that a medium SCHO-to-NDF ratio in the diet enhanced rumen barrier function by promoting claudin-1 expression. In contrast, a high SCHO-to-NDF ratio in the diet disrupted the morphology and integrity of the gastrointestinal epithelium in ruminants and impaired gastrointestinal epithelium barrier function.

The amount of SCHO in the diet has been found to be positively correlated with the amount of LPS in the rumen fluid and the BA content [9,17,20,43]. LPS or other microbe-related molecular patterns can bind to specific pattern-recognition receptors in the gastrointestinal epithelium (such as TLR2 and TLR4) to induce local inflammation [44,45]. A high SCHO-to-NDF ratio in the diet resulted in elevated levels of rumen LPS and increased the mRNA and protein expression of IL-1β and IL-8 [17]. In the present study, we found that a high SCHO-to-NDF ratio in the diet resulted in inflammation in the rumen epithelium, as indicated by increased mRNA levels and concentrations of IL-1β, IL-6, and TNF-α. The results of this study align with the previous findings of Zhang et al. [46] on the rumen of dairy cows fed a high-concentrate diet. Additionally, our previous study revealed that long-term feeding of a high SCHO-to-NDF ratio in the diet increases the concentrations of TNF-α and IL-6 in the blood, leading to an inflammatory response [47]. This result shows that a medium SCHO-to-NDF ratio in the diet did not have a discernible impact on rumen health. However, a high SCHO-to-NDF ratio in the diet can lead to an increase in proinflammatory cytokines and induce rumen inflammation.

The NF-κB and MAPK pathways are the primary inflammatory pathways that mediate LPS stimulation. When LPS binds to the TLR4 complex, IκB phosphorylation leads to the release of NF-κB and a cascade reaction to activate MAPKs, thus promoting inflammatory cytokine expression [13,48,49]. A high SCHO-to-NDF ratio in the diet induces significant changes in the gastrointestinal microbiota of ruminants, resulting in increased numbers of Gram-negative bacteria, increased concentrations of LPS and histamine, and the activation of the NF-κB and MAPK pathway, leading to inflammatory damage in the rumen epithelium [45,50,51]. In this study, we found that feeding a high SCHO-to-NDF ratio diet resulted in a lower pH, a higher LPS concentration, and the increased phosphorylation of p65, IκB, and JNK proteins in the rumen epithelium compared to those in the low-SCHO:NDF group. Under normal physiological conditions, NF-κB and IκB bind in the cytoplasm in an inactivated state. When stimulated by LPS, the phosphorylation and degradation of IκB permit the translocation of NF-κB to the nucleus. This translocation triggers the release of inflammatory cytokines and mediators, leading to inflammatory responses [11,13]. This finding is consistent with the findings of Jiang et al. [52] and Zhao et al. [12,53] in the bovine rumen epithelium. Zhao et al. [12] discovered that SARA caused an increase in LPS levels in the rumen epithelia of dairy cows, thereby overactivating the inflammatory NF-κB and MAPK pathways, which caused the rumen epithelia to release more TNF-α, IL-6, and IL-1β. This result indicates that feeding a high concentration of nutrients may lead to inflammatory reactions in the rumens of lambs by activating the NF-κB and MAPK pathways. In addition, some recent studies have shown that the MAPK pathway can influence changes in tight junctions [54,55]. Zhang et al. [37] reported that a high SCHO-to-NDF ratio in the diet reduced tight junction protein expression and impaired rumen epithelium function through the MAPK pathway. The downregulation of occludin expression in the high-SCHO:NDF group in this study may also be related to the activation of JNK.

## 5. Conclusions

In conclusion, when the SCHO-to-NDF ratio was increased to 1.5, the rumen epithelium was not affected, but when the ratio was increased to 2.0, the NF-kB and MAPK pathways were activated in the rumen epithelium, leading to impaired barrier function and inflammation. The suitable NFC:NDF ratio for the short-term fattening of Dumont lambs was found to be 1.50.

## Figures and Tables

**Table 1:** Composition and nutritional status of the experimental diets (%).

Items	Diets with Different NSC:NDF Levels		
	1.0	1.5	2.0
Ingredients (% of DM)			
*Leymus chinensis*	70.00	50.00	30.00
Corn	28.80	41.00	53.00
Soybean meal	0.00	7.30	14.9
Calcium phosphate dibasic	0.00	0.10	0.10
Salt	0.70	0.60	0.50
Premix1	0.50	0.50	0.50
Sodium bicarbonate	0.00	0.50	1.00
Total	100.00	100.00	100.00
Nutrient levels (%)			
Crude protein	10.70	12.60	14.40
Soluble carbohydrates	37.50	43.60	49.90
Neutral detergent fiber	36.90	30.10	23.30
Calcium	1.40	1.00	0.70
Phosphorus	0.30	0.30	0.30
Acid detergent fiber	27.90	21.50	15.00
Soluble carbohydrates/NDF ratio	1.02	1.45	2.14
Metabolizable energy (MJ/kg)	9.00	9.70	10.40

Provided per kg of premix: Fe 25 mg; Zn 35 mg; Cu 9 mg; Co 0.1 mg; I 0.9 mg; Se 0.25 mg; Mn 19.5 mg; nicotinic acid 60 mg; vitamin E 15 U; vitamin A 3000 U; vitamin D3 1000 U. Metabolizable energy is a calculated value, and the rest of the nutritional indicators are measured values.

**Table 2:** Effects of different soluble carbohydrate-to-neutral detergent fiber ratios in the diet on the growth performance and feed intake of lambs.

Item	Diets			SEM	*p* Value	*P*_linear_	*P*_quadratic_
	L Group	M Group	H Group				
FBW (kg)	31.16 ^b^	37.51 ^a^	35.51 ^a^	0.87	0.004	0.010	0.018
ADG (g)	124.33 ^b^	190.50 ^a^	164.58 ^a^	10.29	0.009	0.050	0.012
ADFI (kg/d)	1.02 ^b^	1.19 ^a^	1.00 ^b^	0.003	0.009	0.669	0.003
SCHOI (g/d)	382.99 ^b^	518.31 ^a^	499.76 ^a^	20.34	<0.001	0.001	0.002
NDFI (g/d)	376.87 ^a^	357.83 ^a^	233.36 ^b^	22.67	<0.001	<0.001	0.006
F/G ratio	8.41	6.42	6.34	0.50	0.177	0.093	0.354

FBW, final body weight; ADG, average daily gain; ADFI, average daily feed intake; SCHOI, soluble carbohydrate intake; NDFI, neutral detergent fiber intake; F/G, feed to gain ratio; data were expressed as mean ± SEM. In the same row, values with different letter superscripts mean significant difference (*p* < 0.05). L group = SCHO-to-NDF ratio was 1.0; M group = SCHO-to-NDF ratio was 1.5; H group = SCHO-to-NDF ratio was 2.0.

**Table 3:** Effect of different soluble carbohydrate-to-neutral detergent fiber ratios in diets on rumen epithelium tight junction mRNA expression in lambs.

Item	Diets			SEM	*p* Value	*P*_linear_	*P*_quadratic_
	L Group	M Group	H Group				
occludin	1.00 ^a^	0.82 ^ab^	0.60 ^b^	0.06	0.004	0.001	0.820
claudin-1	1.00 ^b^	1.93 ^a^	1.53 ^ab^	0.13	0.005	0.021	0.003
ZO-1	1.00	1.04	1.02	0.02	0.756	0.816	0.486

In the same row, values with different letter superscripts mean significant difference (*p* < 0.05).

**Table 4:** Effect of different soluble carbohydrate-to-neutral detergent fiber ratios in the diet on the mRNA expression level of inflammatory factors in the rumen epithelium.

Item	Diets			SEM	*p* Value	*P*_linear_	*P*_quadratic_
	L Group	M Group	H Group				
IL-10	1.00 ^b^	1.50 ^a^	0.84 ^b^	0.09	0.001	0.263	<0.001
IL-1β	1.00 ^b^	1.16 ^ab^	1.44 ^a^	0.07	0.025	0.008	0.647
IL-6	1.00 ^b^	1.82 ^a^	2.08 ^a^	0.17	0.016	0.011	0.344
TNF-α	1.00 ^b^	1.14 ^b^	1.60 ^a^	0.09	0.008	0.003	0.286
IFN-γ	1.00	0.03	1.08	0.03	0.509	0.648	0.293

In the same row, values with different letter superscripts mean significant difference (*p* < 0.05). L group = SCHO-to-NDF ratio was 1.0; M group = SCHO-to-NDF ratio was 1.5; H group = SCHO-to-NDF ratio was 2.0.

## Data Availability

The data presented in this study are available on request from the corresponding author.

## Supplementary Materials

The following supporting information can be downloaded at https://www.mdpi.com/article/10.3390/ani14111666/s1: Table S1: PCR primer sequences of the target genes; Table S2: Antibody types and dilution ratios; Table S3: Effects of different soluble carbohydrate-to-neutral detergent fiber ratios in the diet on rumen epithelial morphology of lambs.

## References

[1] Richeson J.T., Samuelson K.L., Tomczak D.J. (2019). Beef species-ruminant nutrition cactus beef symposium: Energy and roughage levels in cattle receiving diets and impacts on health, performance, and immune responses1. J. Anim. Sci..

[2] Jiang Y., Dai P., Dai Q., Ma J., Wang Z., Hu R., Zou H., Peng Q., Wang L., Xue B. (2022). Effects of the higher concentrate ratio on the production performance, ruminal fermentation, and morphological structure in male cattle-yaks. Vet. Med. Sci..

[3] Plaizier J.C., Mulligan F.J., Neville E.W., Guan L.L., Steele M.A., Penner G.B. (2022). Invited review: Effect of subacute ruminal acidosis on gut health of dairy cows. J. Dairy Sci..

[4] Steele M.A., Greenwood S.L., Croom J., Mcbride B.W. (2012). An increase in dietary non-structural carbohydrates alters the structure and metabolism of the rumen epithelium in lambs. Can. J. Plant Sci..

[5] Wang L., Li Y., Zhang Y., Wang L. (2020). The Effects of Different Concentrate-to-Forage Ratio Diets on Rumen Bacterial Microbiota and the Structures of Holstein Cows During the Feeding Cycle. Animals.

[6] Ogata T., Makino H., Ishizuka N., Iwamoto E., Masaki T., Ikuta K., Kim Y.H., Sato S. (2019). Long-term high-grain diet altered the ruminal pH, fermentation, and composition and functions of the rumen bacterial community, leading to enhanced lactic acid production in Japanese Black beef cattle during fattening. PLoS ONE.

[7] Gozho G.N., Plaizier J.C., Krause D.O., Kennedy A.D., Wittenberg K.M. (2005). Subacute ruminal acidosis induces ruminal lipopolysaccharide endotoxin release and triggers an inflammatory response. J. Dairy Sci..

[8] Guo P., Liu D., Zhao P., Gao M., Hu H. (2015). Effects of Increasing the Ratio of Non-fiber Carbohydrate to Neutral Detergent Fiber on Bacteria Flora, Endotoxin and Histamine Content in Rumen and Plasma of Dairy Goats. Acta Vet. Zootech. Sin..

[9] Ma Y., Wang C., Zhang H., Yu L., Dong L., Gong D., Yao J., Wang H. (2021). Illumina Sequencing and Metabolomics Analysis Reveal Thiamine Modulation of Ruminal Microbiota and Metabolome Characteristics in Goats Fed a High-Concentrate Diet. Front. Microbiol..

[10] Fu Y., He Y., Xiang K., Zhao C., He Z., Qiu M., Hu X., Zhang N. (2022). The Role of Rumen Microbiota and Its Metabolites in Subacute Ruminal Acidosis (SARA)-Induced Inflammatory Diseases of Ruminants. Microorganisms.

[11] Park B.S., Lee J.O. (2013). Recognition of lipopolysaccharide pattern by TLR4 complexes. Exp. Mol. Med..

[12] Zhao C., Liu G., Li X., Guan Y., Wang Y., Yuan X., Sun G., Wang Z., Li X. (2018). Inflammatory mechanism of Rumenitis in dairy cows with subacute ruminal acidosis. BMC Vet. Res..

[13] Sen R., Smale S.T. (2010). Selectivity of the NF-{kappa}B response. Cold Spring Harb. Perspect. Biol..

[14] Wang H. (2020). Research Advances in System-Nutritional Regulation on Mechanism of Carbohydrates Metabolism and Gastrointestin Health of Ruminants. Chin. J. Anim. Nutr..

[15] Zhang L. (2007). Feed Analysis and Feed Quality Detection Technology.

[16] Wang X., Huang J. (2019). Principles and Techniques of Plant Physiological and Biochemical Experiments.

[17] Dai H., Liu X., Yan J., Aabdin Z.U., Bilal M.S., Shen X. (2017). Sodium Butyrate Ameliorates High-Concentrate Diet-Induced Inflammation in the Rumen Epithelium of Dairy Goats. J. Agric. Food Chem..

[18] Gui H. (2020). Effect of Dietary Concentarte Level on RumenEpithelium Growth and Its Underlying Mechanism. Ph.D. Thesis.

[19] Belanche A., Doreau M., Edwards J.E., Moorby J.M., Pinloche E., Newbold C.J. (2012). Shifts in the rumen microbiota due to the type of carbohydrate and level of protein ingested by dairy cattle are associated with changes in rumen fermentation. J. Nutr..

[20] Plaizier J.C., Danesh Mesgaran M., Derakhshani H., Golder H., Khafipour E., Kleen J.L., Lean I., Loor J., Penner G., Zebeli Q. (2018). Review: Enhancing gastrointestinal health in dairy cows. Anim. Int. J. Anim. Biosci..

[21] Kennelly J.J., Robinson B., Khorasani G.R. (1999). Influence of carbohydrate source and buffer on rumen fermentation characteristics, milk yield, and milk composition in early-lactation Holstein cows. J. Dairy Sci..

[22] Han H., Liu D., Gao M., Hu H., Xie C., Deng W., Liu Y., Wang P., Lu D. (2011). Effects of Different Dietary NFC/NDF Ratios on Ruminal Microorganisms and pH in Dairy Goats. Chin. J. Anim. Nutr..

[23] Kleen J.L., Hooijer G.A., Rehage J., Noordhuizen J.P. (2003). Subacute ruminal acidosis (SARA): A review. J. Vet. Med. A Physiol. Pathol. Clin. Med..

[24] Penner G.B., Beauchemin K.A., Mutsvangwa T. (2007). Severity of ruminal acidosis in primiparous holstein cows during the periparturient period. J. Dairy Sci..

[25] Plaizier J.C., Krause D.O., Gozho G.N., McBride B.W. (2008). Subacute ruminal acidosis in dairy cows: The physiological causes, incidence and consequences. Vet. J..

[26] Steele M.A., AlZahal O., Hook S.E., Croom J., McBride B.W. (2009). Ruminal acidosis and the rapid onset of ruminal parakeratosis in a mature dairy cow: A case report. Acta Vet. Scand..

[27] Suárez B.J., Van Reenen C.G., Beldman G., van Delen J., Dijkstra J., Gerrits W.J. (2006). Effects of supplementing concentrates differing in carbohydrate composition in veal calf diets: I. Animal performance and rumen fermentation characteristics. J. Dairy Sci..

[28] Bo Trabi E., Seddik H.-E., Xie F., Wang X., Liu J., Mao S. (2020). Effect of pelleted high-grain total mixed ration on rumen morphology, epithelium-associated microbiota and gene expression of proinflammatory cytokines and tight junction proteins in Hu sheep. Anim. Feed. Sci. Technol..

[29] Ma Y., Wang C., Elmhadi M., Zhang H., Han Y., Shen B., He B.L., Liu X.Y., Wang H.R. (2021). Thiamine ameliorates metabolic disorders induced by a long-term high-concentrate diet and promotes rumen epithelial development in goats. J. Dairy Sci..

[30] Kong L. (2012). The Comparative Study of Calf Stomach Anatomy and Histologicalstructure Development under the Different Feeding Condition. Master’s Thesis.

[31] Otani T., Furuse M. (2020). Tight Junction Structure and Function Revisited. Trends Cell Biol..

[32] Hartsock A., Nelson W.J. (2008). Adherens and tight junctions: Structure, function and connections to the actin cytoskeleton. Biochim. Biophys. Acta.

[33] Sawada N. (2013). Tight junction-related human diseases. Pathol. Int..

[34] Zheng X., Ren B., Gao Y. (2023). Tight junction proteins related to blood-brain barrier and their regulatory signaling pathways in ischemic stroke. Biomed. Pharmacother..

[35] Wei W., Li W., Yang L., Weeramantry S., Ma L., Fu P., Zhao Y. (2023). Tight junctions and acute kidney injury. J. Cell. Physiol..

[36] Markov A.G., Aschenbach J.R., Amasheh S. (2015). Claudin clusters as determinants of epithelial barrier function. IUBMB Life.

[37] Zhang K., Meng M., Gao L., Tu Y., Bai Y. (2019). Rumen-derived lipopolysaccharide induced ruminal epithelium barrier damage in goats fed a high-concentrate diet. Microb. Pathog..

[38] Liu J.H., Xu T.T., Liu Y.J., Zhu W.Y., Mao S.Y. (2013). A high-grain diet causes massive disruption of ruminal epithelial tight junctions in goats. Am. J. Physiol. Regul. Integr. Comp. Physiol..

[39] Lai Z., Lin L., Zhang J., Mao S. (2022). Effects of high-grain diet feeding on mucosa-associated bacterial community and gene expression of tight junction proteins and inflammatory cytokines in the small intestine of dairy cattle. J. Dairy Sci..

[40] Chen M., Xie W., Zhou S., Ma N., Wang Y., Huang J., Shen X., Chang G. (2023). A high-concentrate diet induces colonic inflammation and barrier damage in Hu sheep. J. Dairy Sci..

[41] Han X., Fink M.P., Yang R., Delude R.L. (2004). Increased iNOS activity is essential for intestinal epithelial tight junction dysfunction in endotoxemic mice. Shock.

[42] Gitter A.H., Bendfeldt K., Schmitz H., Schulzke J.D., Bentzel C.J., Fromm M. (2000). Epithelial barrier defects in HT-29/B6 colonic cell monolayers induced by tumor necrosis factor-alpha. Ann. N. Y. Acad. Sci..

[43] Li S., Khafipour E., Krause D.O., Kroeker A., Rodriguez-Lecompte J.C., Gozho G.N., Plaizier J.C. (2012). Effects of subacute ruminal acidosis challenges on fermentation and endotoxins in the rumen and hindgut of dairy cows. J. Dairy Sci..

[44] Kent-Dennis C., Aschenbach J.R., Griebel P.J., Penner G.B. (2020). Effects of lipopolysaccharide exposure in primary bovine ruminal epithelial cells. J. Dairy Sci..

[45] Sun X., Yuan X., Chen L., Wang T., Wang Z., Sun G., Li X., Li X., Liu G. (2017). Histamine Induces Bovine Rumen Epithelial Cell Inflammatory Response via NF-κB Pathway. Cell Physiol. Biochem..

[46] Zhang R., Zhu W., Mao S. (2016). High-concentrate feeding upregulates the expression of inflammation-related genes in the ruminal epithelium of dairy cattle. J. Anim. Sci. Biotechnol..

[47] Ma T., Gao Z., Yang X., Guo S., Li S., Wang H. (2022). Effects of Forage with Varied Concentrate/Roughage Ratios on Growth, Serum Biochemistry, and Immunity of Dumeng Lambs. Fujian J. Agric. Sci..

[48] Lawrence T. (2009). The nuclear factor NF-kappaB pathway in inflammation. Cold Spring Harb. Perspect. Biol..

[49] Zhao C., Wang Y., Yuan X., Sun G., Shen B., Xu F., Fan G., Jin M., Li X., Liu G. (2019). Berberine inhibits lipopolysaccharide-induced expression of inflammatory cytokines by suppressing TLR4-mediated NF-ĸB and MAPK signaling pathways in rumen epithelial cells of Holstein calves. J. Dairy Res..

[50] Sarmikasoglou E., Chu L., Yue F., Faciola A.P. (2023). Effects of ruminal LPS exposure on primary bovine ruminal epithelial cells. J. Dairy Sci..

[51] Pang K., Dai D., Yang Y., Wang X., Liu S., Huang W., Xue B., Chai S., Wang S. (2022). Effects of high concentrate rations on ruminal fermentation and microbiota of yaks. Front. Microbiol..

[52] Jiang M., Wang K., Huang Y., Zhang X., Yang T., Zhan K., Zhao G. (2023). Quercetin Alleviates Lipopolysaccharide-Induced Cell Oxidative Stress and Inflammatory Responses via Regulation of the TLR4-NF-κB Signaling Pathway in Bovine Rumen Epithelial Cells. Toxins.

[53] Zhao C., Yi F., Wei B., Tan P., Huang Y., Zeng F., Wang Y., Xu C., Wang J. (2023). Sodium Propionate Relieves LPS-Induced Inflammation by Suppressing the NF-ĸB and MAPK Signaling Pathways in Rumen Epithelial Cells of Holstein Cows. Toxins.

[54] Naydenov N.G., Hopkins A.M., Ivanov A.I. (2009). c-Jun N-terminal kinase mediates disassembly of apical junctions in model intestinal epithelia. Cell Cycle.

[55] Carrozzino F., Pugnale P., Féraille E., Montesano R. (2009). Inhibition of basal p38 or JNK activity enhances epithelial barrier function through differential modulation of claudin expression. Am. J. Physiol. Cell Physiol..

