# Supplementary material for: The Impact of Different Dietary Ratios of Soluble Carbohydrate-to-Neutral Detergent Fiber on Rumen Barrier Function and Inflammation in Dumont Lambs
Source: Animals (Basel). 2024 Jun 2;14(11):1666. doi: 10.3390/ani14111666 (PMC11171165; doi:10.3390/ani14111666)
Supplement: Supplementary file 1 [file animals-14-01666-s001.zip › animals-2997972-supplementary.pdf]

Table S1. PCR primer sequences of the target genes

Genes	Primer sequence （5'-3'）	Product size/bp	Tm/℃
IL-1β	F：CAGCCGTGCAGTCAGTAA<br>R：TGTGAGAGGAGGTGGAGAG	100	57
IL-6	F：GGGTAAAGAACGCAAAGGT<br>R：TGACCAGAGGAGGGAATG	136	55
IL-10	F：GGCGCTGTCATCGTTTTCTG<br>R：ACACCCCTCTCTTGGAGCAT	91	60
IFN-γ	F：CAGGAGCTACCGATTTCAAC<br>R：AAACCCAAAAGCACACAGA	100	56
TNF-α	F：ACACCATGAGCACCAAAAGC<br>R：AGGCACCAGCAACTTCTGGA	103	60
Claudin-1	F：GTGGATGTCGTGCGTGTC<br>R:TAGTCCCAGCAGGATGCC	127	58
Occludin	F：AGCAGCAGTGGTAACTTGG<br>R：TCCCGTCGTGTAGTCTGTT	111	58
ZO-1	F：CGAGCAGACGCAGAAAA<br>R：GGCAGAAGATTGTGGTTGA	123	55
β-actin	F：CATCGTCCACCGCAAAT<br>R：GCCATGCCAATCTCATCTC	103	56

Table S2. Antibody types and dilution ratios

primary antibodies	dilution ratio	company
Claudin-1	1:1000	Shanghai Beyotime Biotechnology Co.
Occludin	1:1000	Proteintech Group, Inc
Zo-1	1:1000	Proteintech Group, Inc
p65	1:500	Shanghai Beyotime Biotechnology Co.
P-p65	1:300	Beijing Boao Sen Biotechnology Co.
IKB-α	1:800	Shanghai Beyotime Biotechnology Co.
P- IKB-α	1:500	Beijing Boao Sen Biotechnology Co.
P38	1:1000	Shanghai Beyotime Biotechnology Co.

P-p38	1:1000	Shanghai Beyotime Biotechnology Co.
JNK	1:500	Shanghai Beyotime Biotechnology Co.
P-JNK	1:500	Beijing Boao Sen Biotechnology Co.
ERK	1:200	Proteintech Group, Inc
P-ERK	1:200	Affinity Biosciences
β-actin	1:1000	Shanghai Beyotime Biotechnology Co.
secondary antibodies		
IRDye 800CW	1:10000	Li-Cor Inc
IRDye 680RD	1:15000	Li-Cor Inc

Table S3 Effects of different soluble carbohydrate-to-neutral detergent fiber ratios in the diet on rumen epithelial morphology of lambs

Item	Diets			SEM	*P*-value	P_{linear}	$P_{quadratic}$
	L group	M group	H group				
papillae width	157.04	148.48	158.46	2.35	0.177	0.802	0.066
papillae height	899.96^{b}	1336.60^{a}	1358.37^{a}	37.84	<0.001	<0.001	<0.001
papillae area	148791.09^{b}	202029.32^{a}	218595.19^{a}	7261.64	<0.001	<0.001	0.170

Data were expressed as mean ± SEM. In the same row, values with different letter superscripts mean significant difference ($p < 0.05$). L group = SCHO-to-NDF ratio was 1.0; M group = SCHO-to-NDF ratio was 1.5; H group = SCHO-to-NDF ratio was 2.0.
